# Screening for gestational diabetes, Ahmedabad, India

**DOI:** 10.2471/BLT.22.288045

**Published:** 2022-06-22

**Authors:** Himanshu Nayak, Rajendra Gadhavi, Bhavin Solanki, Bhagyalaxmi Aroor, Hemant Gameti, Kalpita S Shringarpure, Jayun Joshi, Zuveriya Kazi

**Affiliations:** a Department of Preventive and Social Medicine, Medical Education Trust Medical College, Ahmedabad, India.; b Department of Preventive and Social Medicine, Byramjee Jeejabhoy Medical College, Ahmedabad, India.; c Ahmedabad Municipal Corporation, Ahmedabad, India.; d Department of Preventive and Social Medicine, Medical College Baroda, Anandpura, Raopura, Baroda, Gujarat, 390001, India.; e Department of Obstetrics and Gynecology, Medical Education Trust Medical College, Ahmedabad, India.; f Gestational Diabetes Mellitus Project, Ahmedabad, India.

## Abstract

**Objective:**

To implement a community-based screening and awareness-raising project for gestational diabetes in Ahmedabad, India.

**Methods:**

The project took place between April 2016 and August 2019 in Ahmedabad. Medical college faculty members and medical officers trained 3582 paramedical staff on screening for gestational diabetes. These paramedical staff tested all pregnant women 24–28 weeks gestation, who were attending village health and nutrition days – also called *mamta* days *–* in urban and rural health centres for routine antenatal care, for gestational diabetes. An oral glucose tolerance test was used and blood sugar ≥ 7.8 mmol/L was the cut-off for gestational diabetes. Women with gestational diabetes were referred for counselling and treatment and all women were followed until 6 weeks after delivery.

**Findings:**

Of 53 522 pregnant women screened, 6786 (12.7%) had gestational diabetes and were referred for nutritional therapy or medication; 836 (12.3%) of these women started medication. There was no significant difference in the prevalence of stillbirths between women with gestational diabetes (0.8%; 54/6786) and women without (0.7%; 338/46 736; *P*-value: 0.51). Of the women on treatment, 38 had abnormal blood glucose after delivery and continued with the medication. Two women with gestational diabetes died; they had other associated co-morbidities – pre-eclampsia and anaemia.

**Conclusion:**

We found a high prevalence of gestational diabetes, indicating the need for gestational diabetes screening and implementation of this project on a larger scale. Gestational diabetes screening at the community level is operationally feasible using the existing human resources and infrastructure of the reproductive health programmes.

## Résumé

**Objectif:**

Mettre en œuvre un projet de sensibilisation et de dépistage du diabète gestationnel au sein de la sphère communautaire à Ahmedabad, en Inde.

**Méthodes:**

Le projet s'est déroulé entre avril 2016 et août 2019 à Ahmedabad. Des médecins et membres de la faculté de médecine ont formé 3582 paramédicaux au dépistage du diabète gestationnel. Ces paramédicaux ont testé toutes les femmes enceintes de 24 à 28 semaines ayant assisté à des journées locales consacrées à la santé et à la nutrition – également appelées journées *mamta* *–* dans les centres de santé urbains et ruraux dédiés aux soins prénatals de routine, afin de détecter un éventuel diabète gestationnel. Ils ont utilisé des tests oraux de tolérance au glucose et fixé la limite à ≥ 7,8 mmol/L pour le taux de glycémie. Les femmes présentant un diabète gestationnel ont ensuite été envoyées en consultation et reçu un traitement; toutes ont été suivies jusqu'à 6 semaines après l'accouchement.

**Résultats:**

Sur 53 522 femmes enceintes dépistées, 6786 (12,7%) souffraient de diabète gestationnel et se sont vu proposer une thérapie nutritionnelle ou des médicaments; 836 (12,3%) d'entre elles ont entamé un traitement. Aucune différence notable n'a été observée en matière de prévalence des mortinaissances chez les femmes atteintes de diabète gestationnel (0,8%; 54/6786) par rapport aux autres (0,7%; 338/46 736; *valeur-p*: 0,51). Sur l'ensemble des femmes sous traitement, 38 présentaient un taux de glycémie anormal après l'accouchement et ont continué à prendre des médicaments. Deux femmes souffrant de diabète gestationnel sont décédées; leur diabète était associé à d'autres comorbidités – prééclampsie et anémie.

**Conclusion:**

Nous avons constaté une forte prévalence du diabète gestationnel, d'où la nécessité de dépister et de déployer ce projet à plus grande échelle. Le dépistage du diabète gestationnel au niveau communautaire est faisable sur le plan opérationnel avec les infrastructures et ressources humaines existant dans le cadre des programmes de santé reproductive.

## Resumen

**Objetivo:**

Poner en marcha un proyecto local de cribado y sensibilización sobre la diabetes gestacional en Ahmedabad, India.

**Métodos:**

El proyecto tuvo lugar entre abril de 2016 y agosto de 2019 en Ahmedabad. Los miembros de la facultad de medicina y los funcionarios médicos formaron a 3582 miembros del personal paramédico en el cribado de la diabetes gestacional. Este personal paramédico realizó pruebas de diabetes gestacional a todas las mujeres embarazadas de entre 24 y 28 semanas de gestación que asistían a las jornadas de salud y nutrición de las aldeas, también llamadas jornadas *mamta*, en los centros sanitarios urbanos y rurales para la atención prenatal de rutina. Se utilizó una prueba de tolerancia oral a la glucosa y se estableció un límite de glucemia ≥7,8 mmol/l para detectar la diabetes gestacional. Las mujeres con diabetes gestacional fueron remitidas para recibir asesoramiento y tratamiento y se realizó un seguimiento de todas ellas hasta 6 semanas después del parto.

**Resultados:**

De 53 522 mujeres embarazadas sometidas a cribado, 6786 (12,7 %) tenían diabetes gestacional y fueron remitidas para recibir un tratamiento nutricional o medicamentos; 836 (12,3 %) de estas mujeres iniciaron la medicación. No hubo diferencias significativas en la prevalencia de mortinatos entre las mujeres con diabetes gestacional (0,8 %; 54/6786) y las mujeres que no la padecían (0,7 %; 338/46 736; valor de *p*: 0,51). De las mujeres en tratamiento, 38 tuvieron una glucemia anormal después del parto y continuaron con los medicamentos. Dos mujeres con diabetes gestacional murieron; tenían otras comorbilidades asociadas: preeclampsia y anemia.

**Conclusión:**

Se detectó una alta prevalencia de diabetes gestacional, lo que indica la necesidad de realizar el cribado de la diabetes gestacional y de aplicar este proyecto a mayor escala. El cribado de la diabetes gestacional a nivel local es factible desde el punto de vista operativo utilizando los recursos humanos y la infraestructura existentes en los programas de salud reproductiva.

## ملخص

**الغرض:**

تنفيذ مشروع للفحص المجتمعي ورفع الوعي بمرض السكري أثناء الحمل في أحمد آباد، بالهند.

**الطريقة:**

تم تنفيذ المشروع خلال الفترة بين أبريل/نيسان 2016، وأغسطس/آب 2019 في أحمد آباد. قام أعضاء هيئة التدريس ومسؤولو الخدمات الطبية في كلية الطب بتدريب 3582 مساعدًا طبيًا، على فحص مرض سكري أثناء الحمل. قام هذا الفريق من المساعدين الطبيين بفحص جميع النساء الحوامل خلال الفترة من 24 إلى 28 أسبوعًا من الحمل، واللاتي كن يحضرن أيام الصحة والتغذية في القرية - وتسمى أيضًا أيام مامتا - في المراكز الصحية الحضرية والريفية للحصول على الرعاية الروتينية السابقة للولادة، للكشف عن مرض السكري أثناء الحمل. تم الاستعانة باختبار لتحمل الجلوكوز الفموي، وكان سكر الدم أكبر من أو يساوي 7.8 مليمول/لتر، هو الحد الأقصى لمرض السكري أثناء الحمل. تمت إحالة النساء المصابات بمرض السكري أثناء الحمل للاستشارة والعلاج، وتمت متابعة جميع النساء حتى 6 أسابيع بعد الولادة.

**النتائج:**

من بين 53522 امرأة حامل خضعن للفحص، كانت 6786 منهن (%12.7) مصابات بمرض السكري أثناء الحمل وتمت إحالتهن إلى العلاج الغذائي أو الأدوية، وبدأت 836 (%12.3) من هؤلاء النساء العلاج بالأدوية. لم يكن هناك فرق واضح في انتشار وفيات الرُضع بين النساء المصابات بمرض السكري أثناء الحمل (%0.8 ؛ 54/6786)، والنساء دون (%0.7 ؛ 338/46736؛ القيمة P للتجانس: 0.51). من بين النساء اللاتي يتلقين العلاج، كان لدى 38 منهن مستوى غير طبيعي من الجلوكوز في الدم بعد الولادة واستمروا في تلقي الأدوية. تعرضت امرأتان مصابتان بمرض سكري أثناء الحمل للوفاة؛ كان لديهما أمراض أخرى، وهي تسمم الحمل وفقر الدم.

**الاستنتاج:**

وجدنا نسبة انتشار عالية لمرض السكري أثناء الحمل، مما يشير إلى الحاجة إلى فحص مرض السكري أثناء الحمل، وتنفيذ هذا المشروع على نطاق أوسع. يعد فحص مرض السكري أثناء الحمل على مستوى المجتمع أمرًا ممكنًا من الناحية التشغيلية باستخدام الموارد البشرية الحالية، والبنية التحتية لبرامج الصحة الإنجابية.

## 摘要

**目的:**

在印度艾哈迈达巴德基于社区实施妊娠期糖尿病筛查和意识提高项目。

**方法:**

2016 年 4 月至 2019 年 8 月期间在艾哈迈达巴德实施该项目。医学院的教职员工和医务人员为 3582 名辅助医务人员提供了妊娠期糖尿病筛查相关培训。这些辅助医疗人员为所有在城乡健康中心参加了“乡村健康和营养日（也称为 *mamta* 日）活动”的 24-28 孕周孕妇提供了妊娠期糖尿病相关常规产前检查。采用口服葡萄糖耐量试验，血糖 ≥ 7.8 毫摩尔/升被视为妊娠期糖尿病的临界值。患有妊娠期糖尿病的孕妇被转诊以便咨询医生和接受治疗，并为所有孕妇在分娩 6 周之前全程提供跟踪随访服务。

**结果:**

在筛查的 53,522 名孕妇中，6786 名（占 12.7%）患有妊娠期糖尿病并被转诊以接受营养治疗或药物治疗；其中 836 名（占 12.3%）开始接受药物治疗。患有妊娠期糖尿病的孕妇 (0.8%; 54/6786) 和未患病孕妇（0.7%; 338/46 736; *P*- 值：0.51） 的死产率并无显著差异。在接受治疗的孕妇中，38 名在分娩后出现血糖异常，进而继续接受药物治疗。两名患有妊娠期糖尿病的妇女最终死亡；因为他们还有其他相关合并症——先兆子痫和贫血。

**结论:**

我们发现妊娠期糖尿病的患病率很高，表明必须扩大开展妊娠期糖尿病筛查和实施该项目的规模。利用生殖健康计划的现有人力资源和基础设施，在社区层面进行妊娠期糖尿病筛查具有可操作性。

## Резюме

**Цель:**

Реализовать проект на уровне местного сообщества по скрининговому обследованию и повышению информированности о гестационном диабете в г. Ахмадабаде, Индия.

**Методы:**

Проект внедрялся с апреля 2016 года по август 2019 года в г. Ахмадабаде. Преподаватели и медицинские работники медицинского колледжа обучили 3582 медицинских работника среднего звена методам скринингового обследования гестационного диабета. Средний медицинский персонал проверил на гестационный диабет всех беременных женщин со сроком беременности 24–28 недель, которые посещали дни здоровья и питания на селе (также называемые днями *мамта*), в городских и сельских медицинских центрах для плановой дородовой помощи. Использовался пероральный тест на толерантность к глюкозе. Уровень сахара в крови ≥ 7,8 ммоль/л был пороговым уровнем для гестационного диабета. Женщин с гестационным диабетом направляли на консультацию и лечение. Все женщины наблюдались в течение 6 недель после родов.

**Результаты:**

Из 53 522 беременных женщин, прошедших скрининговое обследование, 6786 (12,7%) имели гестационный диабет и были направлены на диетотерапию или медикаментозное лечение, а 836 (12,3%) из этих женщин начали прием лекарств. Не наблюдалось существенной разницы в распространенности мертворождений между женщинами с гестационным диабетом (0,8%; 54/6786) и женщинами без гестационного диабета (0,7%; 338/46 736; *P*-значение: 0,51). Среди женщин, которые получали лечение, у 38 после родов наблюдался аномальный уровень глюкозы в крови, поэтому они продолжали принимать лекарства. Две женщины с гестационным диабетом умерли, но у них были другие сопутствующие заболевания: преэклампсия и анемия.

**Вывод:**

Авторы обнаружили высокую распространенность гестационного диабета, что указывает на необходимость скринингового обследования гестационного диабета и реализации данного проекта в более широком масштабе. С оперативной точки зрения скрининговое обследование гестационного диабета на уровне сообщества представляется возможным с использованием существующих кадровых ресурсов и инфраструктуры программ репродуктивного здоровья.

## Introduction

Gestational diabetes mellitus is defined by the American Diabetes Association as, “Any degree of glucose intolerance with its onset or first recognition during pregnancy.”^1^ If not diagnosed and managed adequately, gestational diabetes may have severe life-threatening consequences for the mother, such as pre-eclampsia and obstructed labour, and for the unborn baby, such as preterm birth, macrosomia and shoulder dystocia.^2^^,^^3^ Furthermore, women with gestational diabetes and their offspring are at an increased risk of developing type 2 diabetes later in life.^4^^–^^6^ An estimated 21.1 million live births (16.7% of all live births) in 2021 were affected by hyperglycaemia in pregnancy, and of these, 80.3% were due to gestational diabetes.^7^^,^^8^ Creating awareness of diabetes in general and gestational diabetes specifically, and screening for and prompt management of gestational diabetes, can reduce its worst consequences.^5^^,^^9^ A single initiative of opportunistic screening of women for diabetes during pregnancy can thus have multiple benefits.^7^

Ahmedabad, Gujarat State, has a population of 7 214 225. People in Ahmedabad are at a higher risk of developing diabetes, because of: a preference for high-fat diets; increased access to modern amenities and hence a more sedentary lifestyle; adoption of a westernized lifestyle; and growing urbanization and industrialization. In a 2011 study, the prevalence of type 2 diabetes in Ahmedabad was found to be 13.8% (125/904).^10^ However, no recent estimates of gestational diabetes in women in Ahmedabad or Gujarat are available. No guidelines are available on universal screening for gestational diabetes among pregnant women under the reproductive and child health programme at the state level.^11^ Hence, most women and health-care workers are unaware of these issues.

The government in Gujarat provides diabetes care through noncommunicable disease clinics. Diagnostic facilities are available at community health centres (secondary-care level) and management is undertaken by the general internal medicine department at district hospitals (tertiary-care level).^12^ However, facilities and trained health-care providers are lacking at primary health-care centres and community health centres compared with private health-care facilities that provide services for the management of gestational diabetes. These private facilities are too expensive for low- and middle-income communities.

The Indian government introduced a programme for reproductive, maternal, neonatal, child and adolescent health in February 2013^13^ to improve, among other things, the health of children and women of reproductive age to achieve millennium development goals 4 and 5^14^ as well as the current sustainable developmental goals.^15^ Under the reproductive and child health or the sexual and reproductive health programme in Gujarat, village health and nutrition days – more commonly known as *mamta* days – are routinely held at the village level to provide a basic antenatal care package for pregnant women and immunization for children up to the age of 5 years. *Mamta* days are held on a fixed day every month and at a fixed site, and provide preventive and promotive health services to pregnant and lactating mothers, women of reproductive age (15–49 years), adolescents and children younger than 5 years. Screening for gestational diabetes is not part of routine antenatal care services in India, but identification of gestational diabetes in pregnant women is critical in view of its wide prevalence and the impact it has on pregnancy outcomes. A review undertaken to understand the challenges of and recommendations on gestational diabetes care in India highlighted the need for capacity-building for health-care workers, especially in resource-limited settings.^5^ The review also indicated a need to improve health education among pregnant women on self-management of gestational diabetes.

We report on a pilot project which first trained health-care providers on gestational diabetes and screening during antenatal care, and then used these trained staff to screen for and raise awareness of gestational diabetes among pregnant women attending village health and nutrition days – *mamt*a days – for antenatal care. 

## Methods

### Study design and setting

This was a cross-sectional study with follow-up. Pregnant women between 24 and 28 weeks gestation (the best time to screen for gestational diabetes) attending antenatal care were eligible for inclusion in the project. This project took place in Ahmedabad city (urban) and Ahmedabad district (rural) – population 7.2 million – from December 2016 to April 2019. The project included 72 urban primary health-care centres, 42 rural primary health-care centres, four community health centres and seven medical college hospitals in urban areas and 10 community health centres in rural areas (secondary- and tertiary-care facilities). Health workers screened the pregnant women for gestational diabetes during *mamta* days (every Wednesday) at the urban health centres and primary health-care centres and at antenatal care clinics at the secondary- and tertiary-care facilities. Women found to have gestational diabetes were referred to or further followed up at the community health centres or hospitals.^16^

### Study stages

The study had three stages: (i) training and capacity-building of health-care personnel; (ii) screening of pregnant women for gestational diabetes and raising their awareness of gestational diabetes and diabetes prevention; and (iii) follow-up of the screened women with gestational diabetes for the outcome (pregnancy outcome and treatment needs during and after delivery).

#### Training and capacity-building

We trained medical college faculty members and medical officers so that they could further train paramedical staff (Box 1). The training materials on gestational diabetes were prepared in the local Gujarati language and included information about gestational diabetes, data collection and reporting. The faculty members and medical officers then trained paramedical staff; this training took place in the urban health centres or medical colleges or at the Ahmedabad Municipal Corporation’s offices in urban areas and in the *taluka* (district administrative division) health office. In addition, trained medical officers and staff nurses trained newly recruited paramedical field staff on site (Box 1).

Box 1 **Training sessions and materials on gestational diabetes and topics covered, Ahmedabad, India**

**Training of trainers**

Epidemiology of diabetes; management of diabetes (pharmacological and non-pharmacological); gestational diabetes; gestational diabetes project details (objectives and activities, form for data collection, and monitoring, data recording and reporting formats).

**On-site training of paramedical field staff**

Diabetes: risk factors for diabetes; types of diabetes; symptoms; early detection (screening); preventive measures; and foot care.

Gestational diabetes: explanation of gestational diabetes; causes of gestational diabetes; effect on mother and child; need for management; glucose tolerance testing (video and practical demonstration); information education and communication activities (content, when and how to carry out); and data collection and maintenance of records.

**Information, education and communication sessions and pamphlets for pregnant women**

Scale of gestational diabetes; risk factors for gestational diabetes; effect on mother and fetus; oral glucose tolerance testing; management of gestational diabetes; precautions during delivery and postnatal period; and preventive steps.

**Group discussions or lectures for women during mamta day sessions**

Awareness-raising of gestational diabetes; steps to prevent diabetes in women and their offspring in the future.

#### Screening and awareness-raising

During routine house visits, accredited health workers under the reproductive and child health or the sexual and reproductive health programme identified pregnant women between 24 and 28 weeks gestation and informed them about the objectives of the study. In addition, female health workers identified women when they attended *mamta* days and staff nurses identified women when they attended antenatal care clinics. Trained paramedical staff carried out opportunistic screening of pregnant women for gestational diabetes during the *mamta* days. Similarly, trained staff nurses screened women at secondary and tertiary facilities in urban areas during their routine antenatal visits.

The criteria for gestational diabetes followed the national guidelines,^17^ that is, a single-step test using 75 g of glucose dissolved in 300 mL of water at 24–28 weeks of pregnancy. We also covered pregnant women between 22 and 30 weeks gestation who may have been missed due to the non-availability of screening at 24–28 weeks.

Women were screened irrespective of the time of their last meal and drank the solution within 5 minutes. Blood glucose was evaluated after 2 hours using a standardized glucometer. If vomiting occurred within 30 minutes of glucose load, the test was repeated. However, if vomiting happened after 30 minutes, no retesting was done and the original test was used. A single plasma glucose level ≥ 7.8 mmol/L after 2 hours was the cut-off for gestational diabetes.^18^^,^^19^ The non-response rate was 4.8% (2676/56 198), mainly due to vomiting after taking the 75 g of glucose dissolved in water and declining to take the test again. We only included women who completed the screening test during the analysis (*n* = 53 522).

Health workers used flip charts and information, education and communication videos to educate women of reproductive age and pregnant women about gestational diabetes. They also distributed pamphlets specially designed for the pregnant women who took the oral glucose tolerance test (Box 1).

Representatives of the company that provided the glucometers came regularly to calibrate them. Blood glucose was tested at or after 2 hours, but no later than 2 hours and 15 minutes.^19^ To ensure time accuracy, health workers and staff nurses recorded the time of glucose ingestion and testing in the data collection tool.

We used a three-tier system to monitor the process of screening for gestational diabetes among the pregnant women. Medical officers of health centres attended four sessions at sites within their jurisdiction every month for quality assurance and submitted a quality report to the project office. Similarly, district and state health authorities checked the quality of glucose tests during their routine monitoring. Consultants from the Department of Preventive Medicine and Obstetrics–Gynecology of the medical college carried out external monitoring and submitted a report of their visits to the principal investigators. Repeat testing was done for any invalid tests or if the quality of the data had been affected; however, the numbers were negligible.

Medical officers sent weekly reports based on supervisor registers to monitor progress of gestational diabetes screening. We held review meetings with health supervisors (based on zones in urban areas and *taluka* in rural areas) for the proper documentation, referral of women with gestational diabetes, timely reporting and refresher training.

#### Follow-up

Medical officers counselled women found to have gestational diabetes and referred them to higher centres for appropriate care and delivery. The project followed all women included in the study until delivery or up to 6 weeks after delivery to record their prescribed medications and pregnancy outcomes. Auxiliary nurse midwives followed up the women with gestational diabetes during pregnancy because of locally prevailing customs (women going to their parents’ home after the seventh month of pregnancy to have delivery there) and delivery.

### Analysis

We report frequencies and percentages for the number of women screened, number needing medication and outcomes (successful delivery, stillbirth and treatment needed after delivery). We used a data entry agency which used Excel (Microsoft, Redmond, United States of America) for data entry and verification to minimize error and for data analysis.

### Ethical considerations

The Ahmedabad Municipal Corporation’s Medical Education Trust Ethics Committee provided institutional ethical clearance before starting the project. All the women included in the study gave written informed consent to participate.

## Results

In the training of trainers, 304 faculty members, residents and medical officers received training on teaching paramedical staff about gestational diabetes (Table 1). A further 43 doctors received training during field training of paramedical staff. A total of 3582 paramedical staff received training on gestational diabetes; 2084 (58.2%) were from urban areas and 1498 (41.8%) were from rural areas (Table 1).

**Table 1:** Personnel included in training, Ahmedabad, India, 2016–2019

Personnel trained	No. (%)
**Medical faculty (training of trainers; *n* = 304)**	
Medical college faculty members and residents	76 (25.0)
Medical officers and health officers	228 (75.0)
**Health-care personnel in urban areas (*n* = 2084) **	
Female health workers and auxiliary nurse midwives	621 (29.8)
Accredited social health activists and their facilitators	1306 (62.7)
Laboratory technicians	70 (3.4)
Staff nurses and general nurse midwives	87 (4.2)
**Health-care personnel in rural areas (*n* = 1498) **	
Female health workers and auxiliary nurse midwives	291 (19.4)
Accredited social health activists and others	1169 (78.0)
Laboratory technicians	30 (2.0)
Staff nurses	8 (0.5)

Note: Inconsistencies may arise in some values due to rounding.

Through the information, education and communication campaign, we reached 651 369 women – more than the targeted 300 000 women – with information and material on gestational diabetes. The trained health-care staff screened 53 522 pregnant women for gestational diabetes, more than the target of 50 000 women. Of the women screened, 37 690 (70.4%) were from urban health centres, 14 078 (26.3%) were from primary health-care centres, 1294 (2.4%) were from referral centres and 460 (0.9%) from community health centres. 

Most of the 53 522 women in the study were in the age group 20–30 years (44 937; 84.0%), while 5789 (10.8%) were younger than 20 years, 2791 (5.2%) were 30–40 years of age and five were older than 40 years (0.0%). One third of the women (35.2%; 18 817/53 522) had a primary-school education, 26.6% (14 252/53 522) had a secondary-school education, 16.5% (8851/53 522) were able to read and write, 10.9% (5844/53 522) were educated beyond secondary school and 10.8% (5758/53 522) were illiterate.

Of the 53 522 pregnant women screened, 6786 (12.7%) had gestational diabetes: blood glucose ≥ 7.8 mmol/L (Table 2). They were referred to higher level health-care centres for further diagnosis and management. Of these women, 836 (12.3%) had to start antidiabetes medication. The distribution of the women with gestational diabetes by the type of health centre is shown in Table 2. A greater proportion of women at community health centres and referral centres had gestational diabetes than women at urban and rural health centres.

**Table 2:** Women screened for gestational diabetes, by health centre and test result, Ahmedabad, India, 2016–2019

Type of health centre	Gestational diabetes, no. (%)		Total no.
	Positive	Negative	
Urban health centre	4754 (12.6)	32 936 (87.4)	37 690
Primary health centre	1541 (10.9)	12 537 (89.1)	14 078
Community health centre	121 (26.3)	339 (73.7)	460
Referral centre	370 (28.6)	924 (71.4)	1 294
**Total**	**6786 (12.7)**	**46 736 (87.3)**	**53 522**

Note: Gestational diabetes was defined as pregnant women 24–28 weeks gestation who had blood sugar ≥ 7.8 mmol/L after an oral glucose tolerance test.

Female health workers at the rural centres or staff nurses at the urban centres encouraged all the women to have an institutional delivery. Almost half of the women (49.4%; 26 466/53 522), both with and without gestational diabetes, attended private hospitals for delivery and health care.

Two of the 6786 pregnant women with raised blood glucose died; they had other associated co-morbidities – pre-eclampsia and anaemia. Three deaths occurred among the women without gestational diabetes. Out of 392 stillbirths reported, 54 (13.8%) of the mothers had gestational diabetes, giving a prevalence of 0.8%. Among the women without gestational diabetes, 0.7% (338/46 736) had a stillbirth (*χ*^2^ = 0.43; *P*-value: 0.51).

Of the 836 women on treatment for gestational diabetes, 38 (4.5%) had abnormal blood glucose even after delivery and continued on antidiabetes medication: 14 were on insulin and 24 on orally administered antihyperglycaemic medicines.

Since the screening activity was integrated in the *mamta* sessions and training was done inhouse among current staff in available venues (health centres and corporation buildings), the costs of the pilot project were modest. The main cost was for the glucose powder and glucometer strips which was covered by the project funding agency.

## Discussion

In this pilot project, the trained health workers successfully screened 53 522 pregnant women for gestational diabetes and educated more than 600 000 women about diabetes and gestational diabetes control. The results of the project show that this screening was feasible with the current logistics and contact opportunities with pregnant women and so it could be expanded within the state and later, with supportive evidence, within all India.

*Mamta* days are held every Monday at community and primary health centres and every Wednesday at the community level – as outreach sessions in primary health centre catchment areas and subcentres. They are held 2–3 days a week in urban areas, where reproductive and child health services are provided.^13^^,^^20^ Thus, these days provide a good opportunity to reach the relevant women and children. The plan of the project was to integrate gestational diabetes screening and awareness-raising within the existing reproductive and child health programme. Hence, no separate personnel or infrastructure was needed to implement the project. Health workers (female health workers and accredited social health activists) are the ground staff who are already in contact with the pregnant women during their second trimester (24–28 weeks). Therefore, we can use these opportunities for screening and raising awareness among attendees of health facilities or outreach community camps through *mamta* days.^20^

Furthermore, this screening used the oral glucose tolerance test^4^^,^^8^^,^^18^ at the community level, which is operationally feasible.^21^ Testing the pregnant women in the community 2 hours after taking the oral glucose was not difficult. The prevalence of gestational diabetes among the women screened was 12.7%. This prevalence is higher than that found in studies in rural central India (1.9%; 11/575),^22^ New Delhi (5.7%; 170/2970)^23^ and Haryana (7.1%; 43/607)^24^ but lower than the prevalence in Assam (16.7%; 202/1212).^25^ This higher prevalence indicates the need for implementation of this project on a wider scale during the second trimester, 24–28 weeks. The women who had gestational diabetes were referred and followed up for management and treatment. Diagnosis in this period allows enough time for the health-care provider and pregnant women to control blood glucose levels and reduce the adverse effects of gestational diabetes on mother and baby. Without this pilot project, all the women screened and found positive for gestational diabetes would have gone undiagnosed and untreated. Prompt referral and further management can be a challenge during screening for gestational diabetes.^5^ However, in our pilot project, all the women with gestational diabetes were referred and treated, as accredited social health activists and female health workers undertook routine follow-up of the women for their treatment and delivery. Adherence to medication was also checked and institutional delivery was ensured.

One of the fetal complications of gestational diabetes is stillbirth. As a result of the screening process and the timely referral and management of the women with gestational diabetes, we found no substantial difference in the prevalence of stillbirth among women with gestational diabetes and those without. These rates are lower than the rates found in a study in south India (3%; 4/139 fetal demise among gestational diabetic mothers compared to none in the control group).^26^ Screening for gestational diabetes provides an opportunity to sensitize women about the prevention and control of diabetes through healthy diet and lifestyle and hence reduces the risk of stillbirth.

To achieve the objectives of screening for gestational diabetes and improving awareness and motivation, certain elements are essential, namely: attendance at the right health centre at the right gestational age; local screening; and complete testing and test reporting to allow further management.^27^ Our project had all these elements and hence achieved its objectives.

Both the pregnant women and the health workers responded positively to the screening. Furthermore, the operational feasibility and smooth running of the screening project at the community level show that screening for gestational diabetes can be established as a routine procedure during pregnancy. This project model can be integrated in the reproductive and child health programme, not only in one state but in the whole country, and indeed in other countries that have sound maternal and child health programmes.

Under the Indian National Programme for Prevention and Control of Cancer, Diabetes, Cardiovascular Diseases and Stroke, all primary, secondary and tertiary health centres are well equipped for the diagnosis and management of diabetes. The costs of test material such as glucose powder and glucometer strips were covered by the project funding agency. Any country or region that has an active diabetes control programme and reproductive health programme can replicate this project at little cost, with no additional infrastructure and without placing an extra burden on the health-care personnel. It thus provides a way to reduce maternal and child morbidity and mortality.

After successful completion of the project, an operational feasibility report was submitted to Government of Gujarat. Based on the success of the project, the Maternal and Child Health Division of the Ministry of Health and Family Welfare organized operational training on gestational diabetes through Zoom with relevant stakeholders in India, including state health directors, United Nations Children’s Fund and Jhpiego (formerly Johns Hopkins Program for International Education in Gynecology and Obstetrics) consultants, on 13 December 2019. Ahmedabad city continued gestational diabetes screening activities after completion of the project without external financial assistance. State-level expansion of the project is underway, although it has been delayed because of the coronavirus disease 2019 pandemic.

This study has a few limitations. Since it was a pilot project, it covered only one district and city. Nonetheless, the project succeeded in training many personnel and screening and following up a large number of women using a simple and practical process, which suggests it can easily be extended to the entire country in stages. A structured programme will be required to have a common platform for training the health workers. The glucose powder packages supplied contained 150 g of powder, from which 75 g were used for the oral glucose tolerance test. Later in the study process, we were able to procure 75 g packets after due advocacy. Packages with the exact required quantity would help save time and increase the precision of the dose in routine practice. Certain logistics, such as staff recruitment, training and specific timing of the screening process on specific sessions of *mamta* days, may need to be worked out further, which could be done with liaison with the reproductive and child health programme.
